# The Impact of Bariatric Surgery on Glutathione Synthesis in Individuals with Severe Obesity

**DOI:** 10.3390/antiox13080967

**Published:** 2024-08-09

**Authors:** Hong Chang Tan, Jean W. Hsu, E Shyong Tai, Shaji Chacko, Jean-Paul Kovalik, Farook Jahoor

**Affiliations:** 1 Department of Endocrinology, Singapore General Hospital, Singapore 169608, Singapore; 2 Children’s Nutrition Research Center, Agricultural Research Service, U.S. Department of Agriculture, and Department of Pediatrics, Baylor College of Medicine, Houston, TX 77030, USA; jeanweih@bcm.edu (J.W.H.); schacko@bcm.edu (S.C.); farook.jahoor@bcm.edu (F.J.); 3 Department of Medicine, Yong Loo Lin School of Medicine, National University Health System, Singapore 119228, Singapore; mdctes@nus.edu.sg; 4 Cardiovascular & Metabolic Disorders Program, Duke-NUS Medical School, Singapore 169857, Singapore; jean-paul.kovalik@duke-nus.edu.sg

**Keywords:** glycine deficiency, severe obesity, glutathione synthesis, bariatric surgery

## Abstract

Glycine is deficient in individuals with obesity but improves following bariatric surgery. Glycine deficiency could impair glutathione (GSH) synthesis and worsen oxidative stress. We examined the impact of obesity-associated glycine deficiency and bariatric surgery on GSH synthesis. Twenty-one participants with severe obesity and twenty-one healthy weight controls were recruited. [1,2-^13^C_2_] glycine was infused to measure the erythrocyte (RBC) GSH synthesis rate. Participants with obesity underwent bariatric surgery, and 19 were restudied six months post-surgery. Compared to healthy weight controls, individuals with obesity had significantly lower concentrations of RBC GSH (2.43 ± 0.23 vs. 2.63 ± 0.26 mmol/L, *p* < 0.01). However, there were no differences in GSH fractional synthesis rate [78.0 (51.4–123.7) vs. 76.9 (49.3–110.1) % pool/day, *p* = 0.58] or absolute synthesis rate [1.85 (1.25–3.32) vs. 1.92 (1.43–3.03) mmol/L RBC/day, *p* = 0.97]. Despite a post-surgery increase in glycine concentration, no statistically significant changes in RBC GSH concentration or synthesis rates were detected. Further, the significant correlation between plasma glycine and RBC GSH concentration at baseline (r = 0.46, *p* < 0.01) was also lost following bariatric surgery. GSH concentration was significantly lower in participants with obesity, but bariatric surgery did not significantly increase GSH concentrations or synthesis rates.

## 1. Introduction

Glutathione (GSH) is a tripeptide synthesized from the amino acids glycine, cysteine, and glutamate and is the most abundant intracellular antioxidant and detoxicant in the human body. A decrease in glutathione availability is linked to various cardiometabolic disorders, and erythrocyte glutathione levels are decreased in obesity [1,2]. The lower levels of GSH in individuals with obesity may result from the greater irreversible consumption of GSH relative to its de novo synthesis rate to counter the obesity-induced increased production of oxidants and the toxic byproducts associated with the higher rate of lipid metabolism. Alternatively, GSH biosynthesis may be compromised because of decreased availability of its amino acid precursors.

Amino acid metabolism is deranged in obesity, and studies have consistently reported lower plasma concentrations of glycine but higher concentrations of glutamate and cysteine [3]. We previously showed that slower de novo glycine synthesis in participants with severe obesity resulted in a significantly lower plasma glycine concentration than in healthy weight controls [4]. Since glycine may be rate-limiting for glutathione synthesis [5], the inadequate supply of glycine in obesity may impair GSH synthesis. In the same participants who underwent bariatric surgery, de novo glycine synthesis improved, and plasma glycine concentration increased to values comparable to healthy weight controls six months post-surgery [4]. Thus, if the lower GSH level observed in obese individuals was attributed to glycine deficiency, GSH synthesis should increase after bariatric surgery. Nevertheless, rapid weight loss can impair GSH synthesis despite an adequate supply of its amino acid precursors [6]. Many individuals continue to be in a state of negative energy balance during the first year post-bariatric surgery [7,8], which may explain the variable post-surgery GSH levels reported in the literature. Some studies reported higher post-surgery GSH values [2,9], while others reported that GSH levels decreased [10,11].

This study reports the impact of obesity-associated glycine deficiency on GSH biosynthesis in the same group of participants reported in our earlier study [4]. The stable isotope tracer method was used to measure the rate of GSH synthesis in individuals with severe obesity and normal weight controls. GSH kinetics were re-evaluated six months after bariatric surgery to examine whether GSH synthesis changes with increased plasma glycine concentration. To investigate whether the relationship between glycine availability and GSH was altered by the effects of bariatric surgery, we examined the correlation between plasma glycine and RBC GSH concentrations at baseline and post-surgery.

## 2. Materials and Methods

### 2.1. Participants

We recruited 21 participants with severe (class III) obesity (age between 21 and 65 years and BMI ≥ 32.5 kg/m^2^) from the Singapore General Hospital’s obesity clinic who were scheduled for bariatric surgery. They were excluded if they were receiving insulin treatment, consumed excessive alcohol (>1 drink/day for females or >2 drinks/day for males), received systemic corticosteroid treatment, or had existing end-organ diseases. Twenty-one individuals with a healthy weight (BMI 18.5–24.9 kg/m^2^) were recruited from our healthy volunteer database.

### 2.2. Stable Isotope Infusion

All participants underwent stable isotope tracer studies at baseline. Those in the class III obesity group underwent bariatric surgery and were restudied after six months.

[1,2-^13^C_2_] glycine (99 atom% ^13^C) was used to measure the rate of GSH synthesis according to the tracer infusion protocol previously described [4]. Participants were admitted to the SingHealth Investigational Medical Unit a day before testing. After an overnight fast, baseline samples for metabolite analysis and background isotopic enrichments (IEs) were collected, followed by a primed-constant infusion of [1,2-^13^C_2_] glycine [prime = 8 µmol·kg Fat Free Mass (FFM)^−1^, infusion = 8 µmol·kgFFM^−1^·h^−1^] for the next 7 h. Blood samples were collected hourly from the 4th to 6th hour of infusion and every 15 min during the last hour.

### 2.3. Laboratory Analyses

#### 2.3.1. Glycine and Glutathione Concentration

Plasma glycine concentration was measured using high-performance liquid chromatography, as previously described [4]. The total intracellular GSH concentration was measured in red blood cells (RBCs) by in vitro isotope dilution using [Gly-^13^C_2_, ^15^N]-GSH (Cambridge Isotope Laboratories) as an internal standard. An acetonitrile/water (1:1) buffer was added to RBCs, and then, the samples were “lysed” by freezing and thawing. After centrifugation, the supernatant was transferred and dried. Dithiothreitol (60 mmol/L in 0.1 mol sodium tetraborate/L) was added to convert oxidized glutathione (GSSG) to GSH and was then alkylated by adding iodoacetamide (0.5 mol/L) in 0.1 mol ammonium bicarbonate/L. Alkylated GSH was converted into its DANS [5-(dimethylamino)-1-napthalene sulfonamide] derivative and analyzed using a Kinetex C18 2.6 μ 100 × 2.1 mm column (Phenomenex, Torrance, CA, USA) on a triple quadrupole mass spectrometer (TSQ Altis; Thermo Scientific, San Jose, CA, USA). The ions were then analyzed in SRM mode. The transitions observed were *m*/*z* 598 to 380 and 601 to 380.

#### 2.3.2. Isotopic Enrichments

Isotopic enrichments of glycine and GSH were measured in RBCs using liquid chromatography–tandem mass spectroscopy. Briefly, RBCs were lysed by freezing and thawing. RBC free glycine was converted into its DANS [5-(dimethylamino)-1-napthalene sulfonamide] derivative and analyzed using a Kinetex C18 2.6 μ 100 × 2.1 mm column (Phenomenex, Torrance, CA, USA) on a triple quadrupole mass spectrometer (TSQ Altis; Thermo Scientific, San Jose, CA, USA). The ions were then analyzed in selected reaction monitoring mode. The transitions observed were precursor ions *m*/*z* 309 and 311 to product ion *m*/*z* 170. For GSH isotopic enrichment, the ions were analyzed in SRM mode. The transitions observed were *m*/*z* 598 to 380 and 600 to 380.

#### 2.3.3. Calculation of GSH Synthesis Rate

The fractional (FSR) and absolute (ASR) synthesis rates of RBC intracellular GSH were estimated according to the precursor–product equations:

FSR_GSH_ (% pool/d) = ΔIE_GSH_/IE_RBC_ × 24 (h)

where ΔIE_GSH_ = the isotopic enrichment slope of glutathione (%/h) based on the M + 2 enrichment of glutathione from 0 to 7 h. IE_RBC_ = m + 2 enrichment of RBC free glycine (used as a proxy for intracellular glycine enrichment) during the final hour of infusion.

The ASR of glutathione was calculated as described below and expressed as mmol/L RBC/day:

ASR_GSH_ (mmol/L RBC/day) = FSR_GSH_/100 × intracellular glutathione concentration

### 2.4. Statistical Analysis

The number of subjects that needed to be recruited was estimated based on the ability to detect statistical differences in plasma glycine concentration between the two groups at baseline and in participants with severe obesity after bariatric surgery. Details regarding the power calculation were described previously [4].

The data distribution was examined by plotting a histogram, and its normality was tested using the Shapiro–Wilk test. GSH concentrations had a normal distribution and were presented as mean ± standard deviation. GSH synthesis rates did not follow a normal distribution and were presented as median (interquartile range). The between-group statistical difference in GSH concentration at baseline was determined using the unpaired *t*-test, while the changes in GSH concentration after bariatric surgery were tested using the paired *t*-test. By contrast, the between-group differences in GSH synthesis rates at baseline were tested using the Mann–Whitney test, while the post-surgery changes in GSH synthesis rates were examined using the Wilcoxon signed-rank test. Pearson correlation was used to examine the association between plasma glycine concentration and intracellular GSH concentration at baseline and post-surgery. *p* values < 0.05 were considered statistically significant. Statistical testing was performed using STATA version 17 (Stata Corp, College Station, TX, USA) and Prism version 9 (GraphPad Software Inc., La Jolla, CA, USA).

## 3. Results

**Figure 1:**
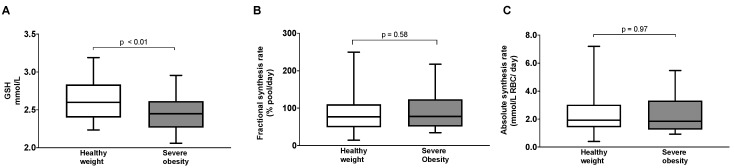
Glutathione concentrations, (**A**) fractional synthesis rates (**B**), and absolute synthesis rates (**C**) in participants with a healthy weight and severe obesity. The between-group difference in GSH concentrations was determined using the unpaired *t*-test, while the differences in GSH synthesis rates were tested using the Mann–Whitney test.

Compared to participants with a healthy weight, those with severe obesity had significantly lower concentrations of RBC GSH (2.43 ± 0.23 vs. 2.63 ± 0.26 mmol/L, *p* < 0.01) (Figure 1A). However, there were no differences in FSR_GSH_ [78.0 (51.4–123.7) vs. 76.9 (49.3–110.1) % pool/day, *p* = 0.58] (Figure 1B) or ASR_GSH_ [1.85 (1.25–3.32) vs. 1.92 (1.43–3.03) mmol/L RBC/day, *p* = 0.97] (Figure 1C).

**Figure 2:**
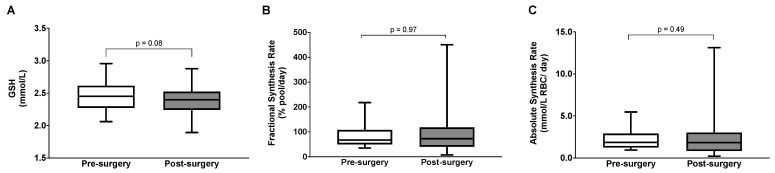
Glutathione concentrations, (**A**) fractional synthesis rates (**B**), and absolute synthesis rates (**C**) in participants with class III obesity before and after bariatric surgery. The significance of post-surgery changes in GSH concentrations was tested using a paired *t*-test, while the Wilcoxon signed-rank test was used for GSH synthesis rates.

Subjects with severe obesity were re-evaluated 6.4 (5.9–8.1) months after bariatric surgery. No significant change in RBC GSH concentration was detected after surgery (2.38 ± 0.27 vs. 2.45 ± 0.23 mmol/L, *p* = 0.08) (Figure 2A). Similarly, there were no post-surgery changes in FSR_GSH_ [72.5 (40.1–117.4) vs. 66.6 (49.4–107.4) % pool/day, *p* = 0.96] (Figure 2B) or ASR_GSH_ [1.83 (0.84–3.02) vs. 1.84 (1.23–2.89) mmol/L RBC/day, *p* = 0.49] (Figure 2C).

**Figure 3:**
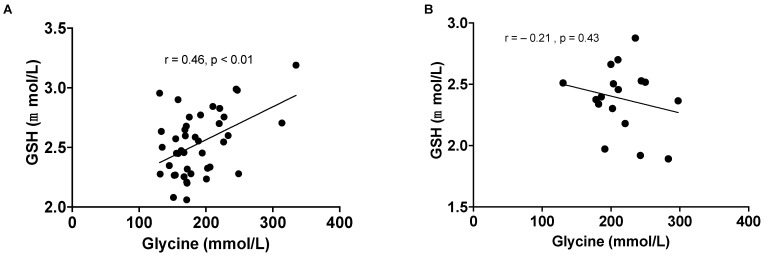
Correlation between plasma glycine and RBC GSH concentrations in all participants at baseline (**A**) and in participants with severe obesity post-surgery (**B**).

At baseline, RBC GSH concentration demonstrated a significant positive correlation with the concentration of plasma glycine (r = 0.46, *p* < 0.01) (Figure 3A). However, this relationship was no longer significant post-surgery (r = −0.21, *p* = 0.43) (Figure 3B).

## 4. Discussion

As a nutritionally non-essential amino acid, diet and endogenous synthesis provide the human body’s daily glycine requirement [12]. In an earlier study in this same group of participants, we reported that endogenous glycine synthesis was compromised in individuals with severe obesity but improved following bariatric surgery [4]. This current report extends our earlier findings by describing the impact of obesity-induced glycine deficiency on GSH synthesis. At baseline, we found a significant correlation between glycine and GSH concentration, and the total GSH concentration in RBCs was significantly lower in participants with severe obesity than in controls with a healthy weight. However, GSH biosynthesis rates were not significantly different. Post-surgery, the relationship between glycine and GSH concentration was lost, and despite greater glycine availability, there were no significant changes in RBC GSH concentration or synthesis rate.

Obesity is considered a state of oxidative stress due to an imbalance between the production rates of pro-oxidants relative to antioxidants such as GSH [13]. It is also associated with an increased production of toxic byproducts associated with a higher rate of lipid metabolism [13]. A sustained supply of GSH is needed to rid the cells of the toxic byproducts of faster lipid catabolism and maintain intracellular oxidative balance and, hence, metabolic function. Consistent with other studies, we found that GSH concentration was significantly lower in participants with severe obesity than in controls [1,2].

Before our study, it was unclear whether the lower GSH levels in individuals with severe obesity were due to impaired GSH synthesis or greater GSH consumption. Our study found that the GSH production rate in participants with severe obesity was not significantly different from that of healthy weight controls. Hence, the lower GSH concentration observed in participants with severe obesity was most likely due to the lack of a compensatory increase in de novo GSH synthesis in the presence of higher irreversible consumption of GSH.

Glycine is regarded as rate-limiting for GSH synthesis [5], and the low glycine supply in this cohort of individuals with severe obesity [4] could explain the lack of the compensatory increase in de novo GSH synthesis. Greater availability of glycine should improve GSH synthesis and raise intracellular GSH concentrations. Indeed, dietary glycine supplementation administered to animals with obesity and humans with chronic HIV infection, old age, and uncontrolled diabetes restored GSH levels and lowered oxidative stress levels [14,15,16,17]. Bariatric surgery also increases the plasma concentration of glycine and reduces oxidative stress levels in individuals with severe obesity [9,18]. Our study found a significant positive correlation between plasma glycine and intracellular GSH concentrations at baseline. Hence, we expected that the increased plasma glycine concentration in this cohort of individuals with severe obesity after bariatric surgery [4] would increase the GSH synthesis rate and intracellular concentration. However, we did not detect significant post-bariatric surgery changes in RBC GSH concentration, GSH FSR, or ASR. We also found that the correlation between plasma glycine and RBC GSH concentration was lost after bariatric surgery.

Glycine availability is usually essential to maintaining GSH synthesis [5]. However, rapid weight loss depresses RBC GSH synthesis in individuals with obesity [6], and we evaluated GSH synthesis six months post-surgery when these individuals consumed about half their baseline calories [4]. Therefore, the state of a negative energy balance post-bariatric surgery likely attenuated the impact of improved glycine supply on GSH synthesis. Our data highlight the complex pathophysiology of redox balance in individuals with severe obesity. More studies are needed before recommending glycine supplementation to individuals with obesity to increase GSH availability, especially when receiving treatment for weight loss.

There are several limitations in this study. This is the first study that reports changes in GSH synthesis in obese individuals following bariatric surgery, but the impact of obesity-induced glycine deficiency on other glycine-dependent pathways also needs to be evaluated. Our study examined changes in GSH synthesis in individuals who recently underwent bariatric surgery. The contemporaneous changes in other amino acids, energy intake, body composition, and physical activity level make it challenging to interpret the relationship between GSH metabolism and glycine availability. Future studies should examine the effectiveness of glycine repletion in individuals with severe obesity independent of changes in body weight. In addition, these studies should be sufficiently large to allow for a gender-specific analysis to evaluate if gender could affect the response to glycine supplementation. Obesity-induced dysregulation in GSH metabolism may be tissue-specific, and we only measured GSH concentration and kinetics in the RBCs. Future studies may consider collecting adipose and liver tissue intraoperatively during bariatric surgery. Nonetheless, low GSH levels in RBCs are believed to reflect GSH depletion in other tissues [19].

## 5. Conclusions

We found a significant correlation between glycine and GSH concentration, and the total GSH concentration in RBCs was significantly lower in participants with severe obesity than in controls with a healthy weight. However, GSH biosynthesis rates were not significantly different. Post-surgery, the relationship between glycine and GSH concentration was lost, and there were no significant changes in RBC GSH concentration or synthesis rate.

## Data Availability

The data described in the manuscript, code book, and analytic code will be made available upon request but subject to approval from the institution’s research office.

## Abbreviations

GSH: glutathione, IE: isotopic enrichment, FFM: Fat Free Mass, RBC: red blood cell, FSR: fractional synthesis rate, and ASR: absolute synthesis rate.
